# CRISPR: fundamental principles and implications for anaesthesia

**DOI:** 10.1016/j.bja.2024.11.040

**Published:** 2025-01-23

**Authors:** Alexendar R. Perez, Orestes Mavrothalassitis, Janice S. Chen, Judith Hellman, Michael A. Gropper

**Affiliations:** 1 Department of Anesthesia and Perioperative Care, University of California, San Francisco, San Francisco, CA, USA; 2 Silico Therapeutics, Inc., San Jose, CA, USA; 3 Mammoth Biosciences, Inc., Brisbane, CA, USA; 4 Department of Physiology, University of California, San Francisco, San Francisco, CA, USA

**Keywords:** chronic pain, CRISPR, critical care, genome engineering, oncology

## Abstract

Clustered regularly interspaced short palindromic repeats (CRISPR)-based medical therapies are increasingly gaining regulatory approval worldwide. Consequently, patients receiving CRISPR therapy will come under the care of anaesthesiologists. An understanding of CRISPR, its technological implementations, and the characteristics of patients likely to receive this therapy will be essential to caring for this patient population. However, the role of CRISPR in anaesthesiology extends beyond simply caring for patients with prior CRISPR therapy. CRISPR has multiple direct potential applications in anaesthesia, particularly for managing chronic pain and critical illness. Additionally, given the unique skills anaesthesiologists possess, CRISPR potentially allows new roles for anaesthesiologists in the field of oncology. Consequently, CRISPR technology could enable new domains of anaesthetic practice. This review provides a primer on CRISPR for anaesthesiologists and an overview on how the technology could impact the field.

**Editor's key points**

• CRISPR is a form of prokaryotic adaptive immunity that is being used to develop novel genetic therapies for inherited diseases and cancer, with potential applications in sepsis and chronic pain.
• CRISPR therapy is now part of modern medicine and will increasingly affect patients cared for by anaesthetists. A fundamental understanding of CRISPR and its potential complications will be essential for effective management of these patients.
• Anaesthetists have the skills to deliver CRISPR therapies targeted to specific tissues such that anaesthesia could be at the forefront of the CRISPR revolution.

CRISPR, which stands for clustered regularly interspaced short palindromic repeats, is a technology that allows modification of an individual's genome.^1^^,^^2^ CRISPR has transformed treatment of genetic diseases as was recently demonstrated with the approval of the world's first CRISPR therapy, Casgevy (exagamglogene autotemcel), to treat sickle cell disease and transfusion-dependent beta thalassemia.^3^^,^^4^ CRISPR clinical trials are currently underway in therapeutic areas including oncology, haematology, cardiology, infectious disease, and rare diseases.^5^ Importantly, the focus of many CRISPR therapies is towards treatments and cures of chronic diseases. Consequently, as CRISPR therapies are introduced into medicine the portion of patients with genetically modified genomes will increase substantially. These patients will inevitably be cared for by anaesthesiologists. However, the role of CRISPR in anaesthesiology is not restricted simply to caring for patients with prior CRISPR therapy. Advances in CRISPR technology have potential direct applications in anaesthesiology, particularly for managing chronic pain and critical illnesses.^6^^,^^7^ Furthermore, CRISPR can enable new roles for anaesthesiologists in the operating room with tumour labelling and destruction.^8^ Anaesthesiologists have traditionally played essential roles in novel therapy development, whether caring for patients in the ICU after cancer immunotherapy, introducing echocardiography for cardiac surgery, or pioneering breakthrough interventional pain therapies.^9^, ^10^, ^11^, ^12^, ^13^, ^14^, ^15^ CRISPR represents a new frontier of medicine and a niche that should be occupied by anaesthesiology.^15^ This review provides a primer on CRISPR for anaesthesiologists and an overview on how the technology could impact the field.

## Basic CRISPR

### CRISPR classification

In nature, CRISPR systems are the adaptive immune system of bacteria and archaea, henceforth termed prokaryotes.^16^ Their purpose is to capture the genetic material of invading viruses and plasmids into a genetic array. This array allows the prokaryote to defend against future infections.^1^^,^^17^ The array encodes effector proteins that function to cleave foreign nucleic acids. These effector proteins also form the basis of how CRISPR systems are classified. Class 1 CRISPR systems utilise multiple proteins to cleave target nucleic acids. Class 1 systems have three subtypes including types I, III, and IV.^18^ Briefly, type I systems are the most common and diverse CRISPR systems in prokaryotes and can unwind and target double-stranded DNA. Type III systems are complex CRISPR systems that can bind and cleave complementary RNA and single-stranded DNA. Type IV systems do not encode DNA or RNA cleaving proteins, but form complexes with small RNAs that generally target plasmids. In contrast, class 2 CRISPR systems use a single effector protein to cleave DNA.^18^ The simplicity of the class 2 system, with only one protein needed to cleave nucleic acids, makes class 2 systems the major focus for therapeutic development. Class 2 systems also have three subtypes including types II, V, and VI.^18^ Type II and V systems both cleave DNA, but type II effector proteins have two nuclease (cleaving) domains while type V effectors have one nuclease domain. Type VI systems target RNA. This review primarily focuses on therapies based on Cas9, a class 2 type II CRISPR system. Cas9 was the first CRISPR protein to be developed as a gene editing therapeutic, and currently is used in the majority of ongoing CRISPR clinical trials, although therapies based on other class 2 CRISPR systems are also rapidly advancing or are in clinical development.^19^

### CRISPR prokaryotic adaptive immunity

**Fig 1:**
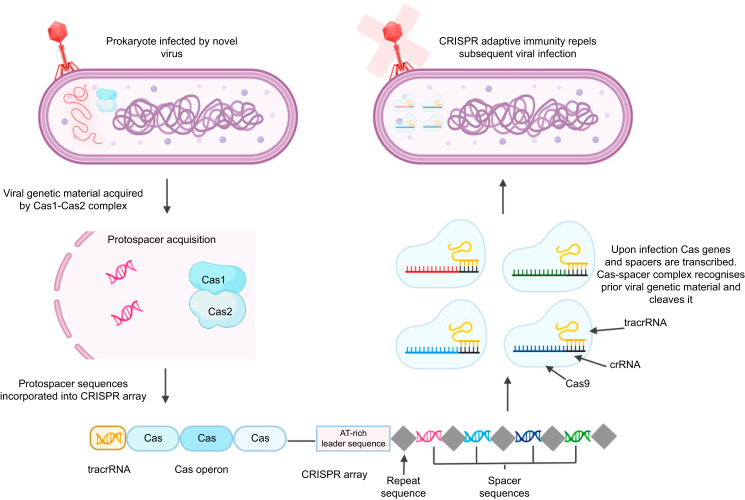
Schematic of type II CRISPR prokaryotic adaptive immunity. Bacteriophage infects prokaryote with viral DNA. Viral DNA is docked by CRISPR-Cas acquisition proteins (Cas1/Cas2) and cleaved to make protospacers. Protospacers are incorporated into the CRISPR array immediately distal to the leader sequence and become spacers. Upon reinfection by virus the CRISPR array is transcribed into a pre-crRNA along with tracrRNAs. TracrRNAs bind to repeat sequences of the pre-crRNA which forms double-stranded RNAs that get cleaved by RNase III. This results in liberated spacer sequences bound to a tracrRNA to form a crRNA-tracrRNA complex that binds with Cas9. The crRNA-tracrRNA-Cas9 complex scans viral PAM sites until a match between the crRNA spacer sequence and the viral DNA occurs. The viral DNA is then cleaved, and the prokaryote repels subsequent viral infection through the CRISPR adaptive immunity mechanism. CRISPR, clustered regularly interspaced short palindromic repeats; PAM, protospacer adjacent motif; pre-crRNA, pre-CRISPR RNA; tracrRNA, transactivating CRISPR RNA.

Prokaryotic genomes are under constant assault from viruses and plasmids. To counteract this threat, prokaryotes evolved an adaptive immune system in the form of a CRISPR array (Fig. 1).^20^^,^^21^ A CRISPR array is composed of CRISPR associated genes (Cas) that are next to an AT-rich segment of DNA called a leader sequence.^22^ The Cas genes encode effector proteins that allow the CRISPR system to target and cleave foreign DNA. Foreign DNA is identified by Cas proteins through a protospacer adjacent motif (PAM). The PAM is a short, stereotyped DNA sequence recognised in viral DNA that allows a Cas enzyme to ensure cleavage occurs against viral, and not prokaryotic, DNA. Once a PAM sequence is recognised, Cas proteins cleave bound genetic material into protospacers.^23^ Protospacers are integrated into the CRISPR array immediately distal to the array's leader sequence, forming a spacer sequence. In between spacers are short palindromic repeat sequences that allow for delineation of spacer sequences.

When a virus infects a prokaryote, the CRISPR array containing spacer and repeat sequences is transcribed into a pre-CRISPR RNA (pre-crRNA)^24^^,^^25^ transcript. Type II CRISPR systems also transcribe a noncoding RNA sequence called a transactivating CRISPR RNA (tracrRNA), which has complementarity to the repeat sequence. Binding of the tracrRNA to the pre-crRNA repeat sequences generates a double-stranded RNA (dsRNA) substrate that undergoes further processing by RNase III. This results in tracrRNAs and mature crRNAs that contain a spacer sequence from a prior infection. The tracrRNA, crRNA, and Cas protein form a complex that binds and cleaves invading viral DNA resulting in prokaryotic adaptive immunity.^26^

In the context of Cas9 genome engineering, the crRNA and tracrRNA are synthetically fused to make a single guide RNA (sgRNA, or more generally, gRNA).^27^ The gRNA contains a 20-nucleotide targeting element that determines where a CRISPR endonuclease binds, and ultimately cuts, in a genome.^27^ CRISPR gRNAs and Cas9 form a complex that searches and docks target DNA. Target cleavage occurs when 1) the CRISPR endonuclease recognises a PAM sequence and 2) sufficient complementarity exists between the 20-nucleotide 5′ end of the gRNA and the target DNA.^28^, ^29^, ^30^ Notably, Cas9 tolerates mismatches between the gRNA and target DNA, which allows for off-target cleavage in non-target sites across the genome.^31^^,^^32^

## CRISPR technologies

Genome engineering allows permanent modification of DNA sequences. However, until recently, the process has been time-consuming and labour intensive because of the limitations of available technologies. The discovery of CRISPR and the repurposing of its Cas proteins as genetic engineering tools has reshaped the trajectory of genome engineering because of their simplicity and ease of use.^27^^,^^28^ By simply changing the sequence of a gRNA targeting region, endonucleases such as Cas9 can be redirected to any site in the genome that is adjacent to a PAM sequence. Traditional CRISPR-based methods generate site-specific DNA double-strand breaks (DSBs) and rely on cellular DNA repair pathways to achieve genome modification.^33^ However, newer CRISPR-based technologies enable precise and versatile genome engineering without the need for DSBs. These non-DSB technologies include base editing, prime editing, epigenetic modulation, and RNA editing.

### Genetic editing with double-strand breaks

**Fig 2:**
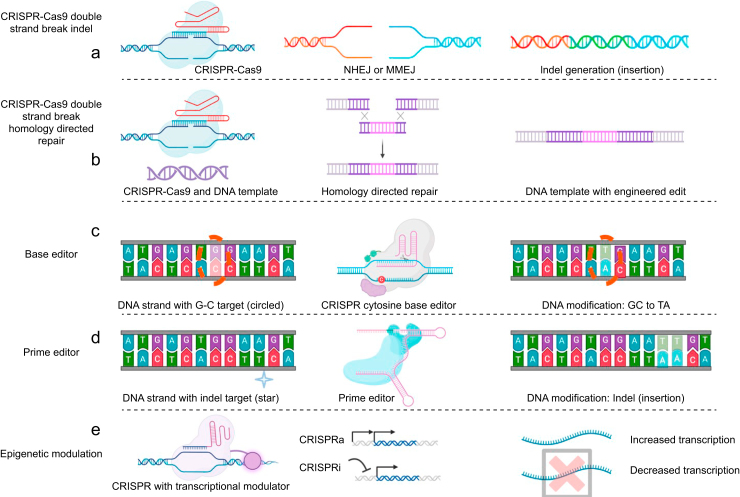
CRISPR technologies. (a) CRISPR-Cas9 DNA double-strand break (DSB)-mediated gene knockout. The CRISPR-Cas9 complex docks target DNA and induces a DNA DSB. When no DNA template is present the repair occurs through non-homologous end joining (NHEJ) or microhomology-mediated end joining (MMEJ). This generally results in the formation of insertions or deletions (indels), leading to gene knockout. (b) CRISPR DSB with homology-directed repair (HDR)-mediated modification. CRISPR-Cas9 induces a DNA DSB with a DNA template present, which uses DNA homology to repair the DSB. This incorporates the engineered template into the genome. (c) Base editors allow the conversion of target C-G DNA pairs to T-A DNA pairs with cytosine base editors and the A-T DNA pairs to G-C DNA pairs with adenine base editors. This is achieved by deaminase domains fused to a dCas9 or Cas9 nickase. (d) Prime editors allow all base modifications and introduce small indels. Prime editors utilise a Cas9 nickase fused with a reverse transcriptase domain and a prime editing guide RNA (pegRNA) to enable these modifications. (e) CRISPR gene activation using the transient CRISPR activation (CRISPRa) or durable CRISPRon activation, and transient CRISPR inhibition (CRISPRi) or durable CRISPRoff inhibition epigenetic modulation methods. These methods use dCas9 fused to transcriptional activation or repression domains to affect expression of target genes or DNA loci. CRISPR, clustered regularly interspaced short palindromic repeats.

The ability of CRISPR to induce DNA DSBs at a desired genomic location forms the basis of its utility as a genome engineering technology. CRISPR enables modification of a target genome by invoking DNA repair responses to DSBs. The most common repair mechanism for DSBs is the non-homologous end joining (NHEJ) pathway (Fig. 2a).^34^^,^^35^ During NHEJ the two ends of the recently cut DNA are functionally rejoined.^34^ However, repair by NHEJ can lead to a loss of genetic information either from deletion or insertion (indel) of genetic material at the site of the DSB. Less commonly, DSBs can be repaired by microhomology-mediated end joining (MMEJ), which deletes genetic information on both sides of the DSB until microhomologies are present.^36^ MMEJ uses these microhomologies to align the cut segments and repair the DNA. This process can introduce indels that are often larger than those seen in NHEJ.^37^ If an indel occurs in the coding sequence or intron splice sequences of a gene, it can disrupt or knock out the gene.^38^ Consequently, CRISPR can be used to inactivate DNA sequences through targeted indel generation. However, generation of DSBs can lead to potential undesirable genotoxicities such as chromosomal translocations or genomic instability.^39^

CRISPR can also be used to replace genetic material through homology-directed repair (HDR) (Fig. 2b).^40^ In this setting either a single-stranded or double-stranded DNA molecule is delivered to a target cell with the CRISPR system. This DNA molecule contains a specific change introduced at the target site, and sequences with homology to the insertion site. When the CRISPR complex induces a DSB, the DNA molecule acts as a template for repair and the modification is incorporated into genomic DNA, with a few caveats. Firstly, HDR is an inefficient process compared with NHEJ; most DSBs will be repaired with NHEJ.^41^ Secondly, HDR is active when cells are dividing, but is relatively inactive when cells are not dividing.^42^ In contrast, NHEJ is always active.^43^ Improving the rate and efficiency of HDR is an area of active investigation.^44^

### Genetic editing without double-strand breaks

The Cas9 protein has two nuclease domains; inactivation of one or both of these domains generates versions of Cas9 that can bind to target DNA without cutting DNA (dCas9) or that cut only a single strand of DNA (nickase).^45^^,^^46^ Cas9 nickase is the most widely utilised scaffold that enables base editing and prime editing technologies, which modify target DNA without inducing DSBs.

CRISPR base editors utilise either a Cas9 nickase or dCas9 to bind to target DNA (Fig. 2c).^47^, ^48^, ^49^ Once bound, the segment of DNA containing the PAM sequence becomes displaced as a single strand of DNA. Base editors have deaminase domains fused to the Cas9, and when the single-stranded DNA is exposed, it is deaminated by the deaminase.^50^ CRISPR base editors can catalyse CG-to-TA conversions with cytosine base editors and AT-to-GC conversions with adenine base editors.^50^^,^^51^ This allows CRISPR base editors to potentially correct pathogenic mutations without the concerns of inducing a DSB in a target genome. However, the editing window is limited to a few nucleotides and these modifications only account for a subset of all possible base-to-base changes.

CRISPR prime editors can alter single nucleotides with all possible base changes and introduce small indels into target DNA without donor DNA templates (Fig. 2d).^52^ Prime editors use a Cas9 nickase, a reverse transcriptase domain, and a prime editing gRNA (pegRNA) to achieve this.^53^^,^^54^ Prime editors use their pegRNA to bind both strands of DNA, but only cut one strand.^53^^,^^54^ The pegRNA has sequence complementary to the strand that is cut and a 3′ extension including the desired alteration. The extension is reverse transcribed resulting in a DNA–RNA pair containing the edit.^53^^,^^54^ Subsequent repair processes incorporate the alteration permanently into DNA at a set frequency. This technology can potentially reverse deleterious mutations whether they exist as single base mutations or frameshift mutations resulting from pathogenic indels. However, prime editing is limited to short sequence insertions (<50 base pairs) with low efficiency for larger insertions.

### Epigenome and transcriptome engineering

CRISPR can increase or decrease the expression of genes through epigenetic modulation (Fig. 2e). Epigenetics regulates gene expression from the transcriptional to post-translational level.^55^ CRISPR activation (CRISPRa) and CRISPR inhibition (CRISPRi) are methods that augment or inhibit transcription at a genetic locus, respectively.^56^^,^^57^ CRISPRa works by fusing transcription activation domains to a dCas9 which results in the recruitment of transcriptional machinery and increased expression of a genetic locus.^56^ In contrast, CRISPRi brings transcription repressive domains to a locus which inhibits expression of the site.^57^ Epigenetic modulations using CRISPRa/CRISPRi are generally short-lived, though longer-term silencing and reactivation are possible with the CRISPRoff and CRISPRon versions of the system.^58^ Overall, this technology holds promise for allowing interventions on epigenetic causes of disease but is limited to applications that involve silencing or activating genes.

CRISPR can also modify RNA. This typically involves an inactivated version of a class 2 type VI CRISPR system called dCas13 fused with the RNA specific deaminase system ADAR2.^59^ The process starts when dsRNA gets bound by the dCas13 gRNA. The gRNA carries the desired edit inside its complementary sequence which results in mismatches between the RNA and the gRNA.^59^^,^^60^ These mismatches trigger ADAR2 allowing editing of the RNA.^59^^,^^60^ This technology could correct mutated gene products without the need to alter the genome itself. However, RNA editing is highly transient and would likely require repeat dosing to achieve the desired therapeutic effect.

## Clinical CRISPR and its limitations

A key advantage of CRISPR genome engineering is its potential as a curative therapy for genetic causes of disease. There are currently on-market genetic medicines for several indications using modalities such as traditional gene therapy, antisense oligonucleotides (oligos), and small interfering RNA (siRNA), but they are all limited by durability of effect. For gene therapy, the vector carrying the gene replacement gets diluted out of target tissues over time, and because of immunogenicity concerns, the therapy generally cannot be re-dosed.^61^, ^62^, ^63^ Antisense oligos and siRNAs, however, require repeat dosing to achieve durability, and the dosing schedule can range from every few weeks to several times a year over a patient's lifetime to maintain therapeutic benefit.^64^, ^65^, ^66^

**Fig 3:**
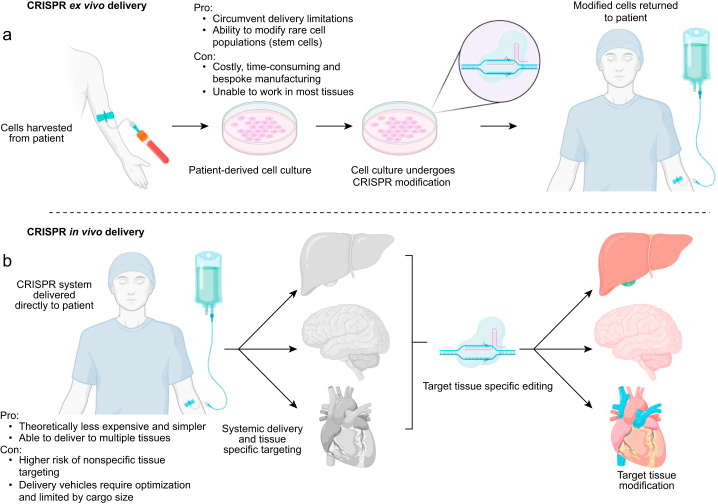
CRISPR delivery paradigms. (a) *Ex vivo* delivery with CRISPR modification occurring outside of an individual with edited cells delivered back to patient. Cells are taken from the patient and modified *in vitro* before being given back to the patient. (b) *In vivo* delivery with CRISPR modification occurring at target tissue within a patient. In this paradigm, CRISPR systems are packaged through viral vectors or lipid nanoparticles to allow for tissue-based or systemic delivery. Genomic modification occurs at the target tissue so tissue target specificity is a critical concern. CRISPR, clustered regularly interspaced short palindromic repeats.

For CRISPR to work as a medicine, the genome engineering machinery must reach the target cells and tissues, which can be accomplished through *ex vivo* or *in vivo* delivery. For *ex vivo* therapies, genetic engineering takes place outside the body and involves extracting stem cells, modifying the cells in a laboratory, and infusing modified cells back into the patient (Fig. 3a).^67^^,^^68^ This process is complex and involves bespoke manufacturing and the requirement that the patient undergo chemotherapy. The entire treatment can take up to 1 yr.^69^^,^^70^ However, *ex vivo* therapies circumvent the challenges of *in vivo* delivery and are a promising approach to create functional cures for certain haematologic disorders and cancers, several of which are being evaluated in clinical trials.^5^ For *in vivo* editing, genome engineering takes place inside the body (Fig. 3b). Unlike blood, cells from other tissues such as muscle and brain cannot be extracted for *ex vivo* editing, and therapeutic approaches for a vast number of genetic diseases require targeted *in vivo* delivery of genome engineering components. *In vivo* editing can be delivered locally to a specific organ or systemically through i.v. delivery, a process that is much simpler and more cost-effective than *ex vivo* editing.^71^, ^72^, ^73^ There are several ongoing clinical trials for *in vivo* CRISPR therapies, although none have yet reached regulatory approval.^71^^,^^74^

Despite the expanded capabilities of CRISPR-based technologies, *in vivo* delivery of CRISPR gene editors to specific tissues remains an ongoing challenge. There are two types of clinically validated delivery technologies that can carry CRISPR cargo, which include non-viral methods that target the liver (e.g. lipid nanoparticles [LNPs], the technology used to deliver mRNA for COVID-19 vaccines^75^^,^^76^), and viral methods that target tissues beyond the liver including muscle, heart, brain or lung (e.g. adeno-associated viruses [AAVs], which are non-pathogenic viruses that have been modified to deliver DNA to target cells).^77^, ^78^, ^79^, ^80^ Both LNPs and AAVs show tremendous promise for systemic *in vivo* CRISPR delivery. LNPs are currently being tested as a delivery vehicle in clinical trials and AAVs are quickly following suit. Despite their promise, both delivery modalities have limitations. For example, LNPs can package mRNAs encoding large CRISPR machinery and have preferential delivery to the liver, but tissue-specific delivery beyond the liver is a challenge. AAVs have tropism for extrahepatic tissues such as muscle, heart, brain, and lung, but have limited cargo capacity, and large CRISPR enzymes such as Cas9 cannot fit within a single AAV vector.^81^, ^82^, ^83^ However, ongoing efforts to decrease cargo size using smaller CRISPR systems, such as Cas14 (Cas12f) or CasPhi (Cas12j), that fit within a single AAV have expanded the possibilities of using AAV vectors to deliver genetic editing technologies to specific extrahepatic tissues.^84^, ^85^, ^86^, ^87^, ^88^

### CRISPR safety

**Fig 4:**
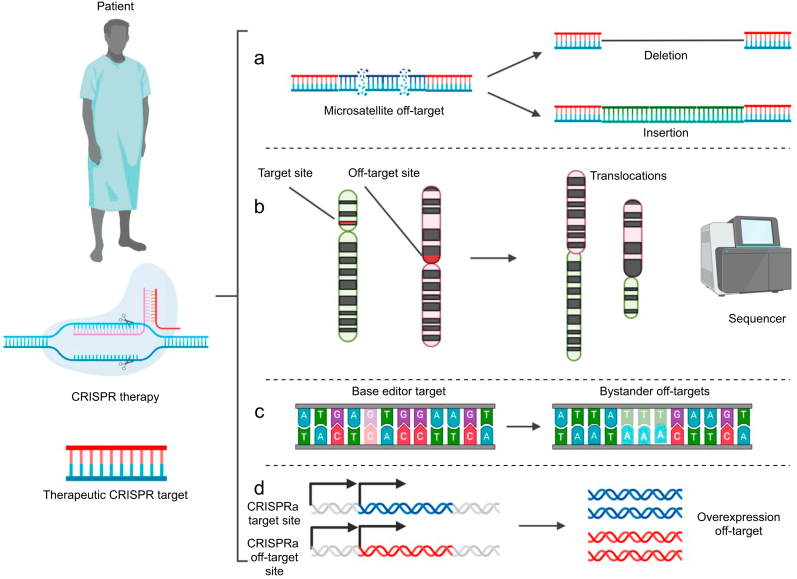
CRISPR off-target sites. (a) Unintentional indel generation resulting from nonspecific CRISPR guide RNA (gRNA) target. This can result from gRNAs with multiple exact targets or targets that are dissimilar by a few positional mismatches between a gRNA and target DNA. A potentially disruptive type of off-target effect is the unintended targeting of repetitive loci that can generate indels and genomic instability. (b) Chromosomal translocations following nonspecific gRNA design. This can occur if a gRNA has a minimum of two effective cleavage targets on distinct chromosomes. (c) Bystander mutations from base editors occur when bases outside the intended target are edited by the base editor. Here the desired target is in the left fade and the actual edit is in the right fade. (d) Unintended off-target transcriptional upregulation resulting from a nonspecific CRISPR activation (CRISPRa) gRNA. Transcriptional activation domains are misdirected to an undesired gene locus resulting in overexpression of an off-target gene. CRISPR, clustered regularly interspaced short palindromic repeats.

An essential consideration for all CRISPR medicines is their safety profile, and particularly their tissue and genomic target specificity. CRISPR off-target effects occur when non-target nucleic acids are modified by a CRISPR system.^89^ All CRISPR-based technologies have the potential to generate off-target effects that can result from poor gRNA target specificity. With CRISPR-mediated DSBs, off-target DSBs can result in translocations, indels, and genomic instability (Fig. 4a and b).^89^^,^^90^ With CRISPR base editing technologies, mutations can occur both at the target site and nearby nucleic acids around the target site to introduce bystander modifications (Fig. 4c).^91^^,^^92^ Furthermore, base editing technologies can modify single-stranded DNA by gRNA-independent off-target modification.^93^

Prime editors have fewer off-target effects than base editors, but have limited proof-reading capability and can incorporate tracrRNA scaffolds into a genome through excessive reverse transcriptase activity.^94^ With epigenetic modifications, off-target effects can lead to undesired genetic expression changes that can affect cellular fitness (Fig. 4d).^95^ Off-target effects in RNA are of lesser concern, given their transient nature, though they too can lead to alterations in non-target RNAs that could disrupt their translation or function.^96^, ^97^, ^98^, ^99^ Consequently, the proper and rational design of gRNAs is an essential part of ensuring the safety of any CRISPR-based therapy.

There are multiple approaches that can minimise off-target potential, including but not limited to targeted delivery, high-fidelity Cas endonucleases, selection of gRNAs that are known to have minimal predicted off-targets in the human reference genome, chemical gRNA modifications, and experimental confirmation of potential off-target sites in a biologically relevant context.^100^, ^101^, ^102^, ^103^ While any two individuals are ∼99.9% identical genetically, about one of every 1000 bases will differ between them.^104^ At the scale of the human genome, any two individuals will have ∼6 000 000 base differences between them.^104^ In a study of the role of human genetic variation in gRNA off-targets, 7444 human whole genome sequences were analysed to determine how single-nucleotide polymorphisms (SNPs) and individual level indels contributed to Cas9 gRNAs off-target universes.^105^ Individual level genetic variation created ∼11 500 000 new PAM sites (4.1% of PAM sites) and eliminated ∼22 000 000 PAM sites (7.9% of PAM sites).^105^ At the population level, one of 10 PAM sites can be either nonexistent (ablated reference genome PAMs) or a potential unexpected off-target site (individual genome *de novo* PAMs). Consequently, for CRISPR gRNAs designed off the human reference genome, off-target effects from human genetic variation can be a concern for therapeutic development. Given that CRISPR directly acts on the genome, designing gRNAs that are specific to an individual can offer powerful approaches for achieving safety in a genome-specific context.

## Anaesthesia for the CRISPR patient

### Casgevy

In 2023, Casgevy® became the first CRISPR-based therapeutic when it was approved by the US Food and Drug Administration and European Medicines Agency for the treatment of sickle cell disease and transfusion-dependent beta-thalassaemia.^3^ Casgevy uses CRISPR to target the erythroid-specific enhancer region of the BCL11A gene^106^^,^^107^ which encodes a transcription factor that mediates the switch from production of fetal haemoglobin to adult haemoglobin. By reducing BCL11A expression, Casgevy increases levels of fetal haemoglobin. This greatly reduces the symptoms and pathology of sickle cell anaemia and beta-thalassaemia, as these diseases result from mutations in adult haemoglobin and not fetal haemoglobin.^108^ Casgevy treatment costs ∼2.2 million USD per dose, and the start of therapy to the end of therapy is up to 1 yr.^109^, ^110^, ^111^ Lifelong medical therapy for sickle cell disease and its complications including hospital admissions for vaso-occulsive episodes is estimated to cost 1.6–1.7 million USD with an average out of pocket expenditure of 44 000 USD per patient.^112^

Casgevy manufacture involves apheresis and isolation of CD34+ haematopoietic stem and progenitor cells (HSPCs) from patients with sickle cell disease or beta-thalassaemia. Next these cells undergo CRISPR-Cas9 editing at the BCL11A erythroid-specific enhancer region to increase production of fetal haemoglobin. Afterwards, the patient undergoes myeloablative conditioning with busulfan followed by i.v. administration of the edited HSPCs for durable engraftment. The therapy leads to increased levels of fetal haemoglobin in treated sickle cell and beta-thalassaemia patients leading to significantly decreased symptom burden and enhanced quality of life. Importantly, higher levels of fetal haemoglobin translate to a left shift in the oxyhaemoglobin dissociation curve for these patients.^113^ Consequently, their haemoglobin has greater affinity for oxygen and reduced oxygen unloading capacity. Thus these patients might require longer pre-oxygenation times before induction of anaesthesia, and could be candidates for permissive hypercapnia to allow greater oxygen unloading to prevent relative tissue hypoxaemia. This novel class of therapy is a result of >35 yr of basic science research spanning domains including bacterial genetics, basic biochemistry, and human immunology.^114^^,^^115^ Casgevy reinforces the value of fundamental research to the advancement of clinical medicine.

### CRISPR in the perioperative and critically ill patient

**Fig 5:**
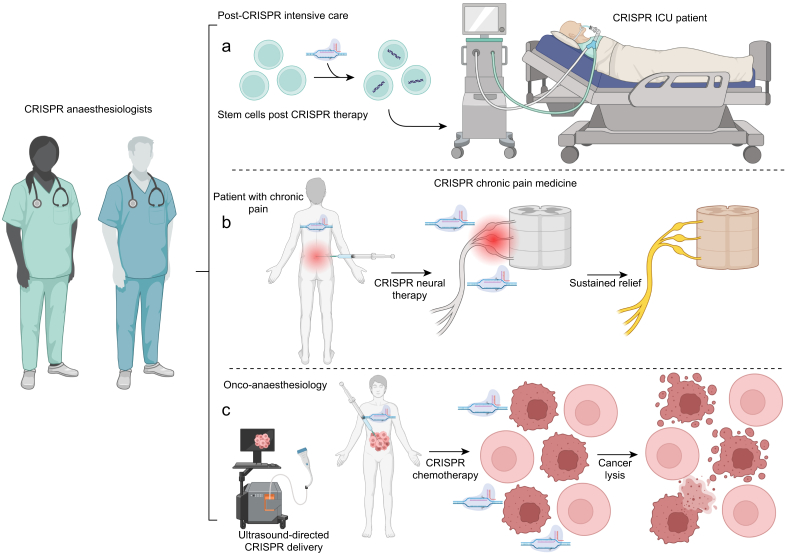
CRISPR in Onco-anaesthesiology. (a) ICU settings are prime areas where anaesthesiologists both care for CRISPR patients and potentially utilise CRISPR-based therapy for diagnosis and treatment of critical illness. Use of CRISPR immunomodulators for sepsis is one potential application of CRISPR in the ICU that is under active development. (b) CRISPR therapies that transiently or permanently alter genetic pathways implicated in pain. Neuraxial and regional anaesthesia techniques potentially allow tissue-specific delivery of CRISPR therapeutics. (c) CRISPR as a direct anticancer modality could be delivered in a tissue-specific manner using neuraxial or regional anaesthesia techniques using ultrasound technology. CRISPR, clustered regularly interspaced short palindromic repeats.

With the approval of Casgevy, it is anticipated that anaesthetists will soon be caring for patients who have undergone CRISPR treatments (Fig. 5a). The first CRISPR clinical trials are focused on haematologic and oncologic disorders, congenital disorders, and infectious diseases.^116^^,^^117^ Because of the inherent risk of a novel therapeutic class, initial trials have generally targeted patient populations refractory to first-line or traditional therapies, and these patients generally have advanced disease. For example, the CRISPR therapeutic NTLA-2001 is currently being evaluated in a multicentre international phase III clinical trial among patients with transthyretin amyloidosis, cardiomyopathy, and heart failure, with the primary outcome being a composite of cardiovascular mortality and cardiovascular events.^71^^,^^118^ CRISPR therapies are also being used to produce chimeric antigen receptor (CAR) T cells targeting CD-7 for treatment of relapsed or refractory T-cell acute lymphoblastic leukaemia or lymphoma (drug BEAM-201, phase I/II clinical trial), and CD-19 for the treatment of relapsed or refractory B-cell malignancies and autoimmune disease (drug CTX112, phase I/II clinical trial).^119^, ^120^, ^121^ As such, anaesthetists caring for perioperative or critically ill patients receiving investigational or newly approved CRISPR-based therapies should be aware that these patients will likely have a sequelae of advanced underlying disease (heart failure, tumour burden, poorly controlled glucose, etc.). They may also suffer from side-effects of past failed therapies (anaemia, pulmonary toxicity, hormonal dysregulation, and immune suppression).

Patients receiving CRISPR therapies might also experience complications from either genome engineering itself or systemic adverse reactions to the vehicles used to deliver genome engineering therapies. As a result, there is an effort to minimise the risk of off-target effects from CRISPR. Specific techniques include *in silico* tools to predict the risk of off-target guide RNA interactions and cell-free and cell culture methods to directly sequence *ex vivo* edited cell lines before administration to patients.^122^, ^123^, ^124^, ^125^ Studies of the first patients to receive Casgevy have found no evidence of off-target effects, although these studies will have to be repeated at scale as patients continue to receive gene editing therapies.^126^ Such off-target effects would be expected to be haematologic or oncologic in nature, and would likely not manifest until years after the CRISPR editing event.

The most significant risks to perioperative and critically ill patients will likely be the side-effects and systemic reactions resulting from the delivery methods and vehicles of CRISPR therapies. As an example, therapies to edit haematopoietic stem cells (e.g. for treatment of cancers or haematologic disorders) generally involve lymphodepletion or myeloablative therapy before administration of CRISPR *ex vivo* edited cells. When Casgevy was administered to both sickle cell and beta-thalassaemia patients, the rate of grade 3 or 4 adverse events was 95% and 88%, respectively.^106^^,^^127^ The adverse events most common and germane to anaesthesia care were stomatitis (55% and 40%, respectively), febrile neutropenia (48% and 54%, respectively), and thrombocytopenia (25% and 35%, respectively), with their resultant implications for pain control, antibiotic management, and regional anaesthesia.^106^^,^^127^ These reactions might also result from the vehicles used to deliver CRISPR editing machinery, including AAVs and LNP.^71^ While AAVs are not profoundly immunogenic, they can activate both the innate immune system and the complement system, leading to rare events, including but not limited to cytokine storm, hepatotoxicity, and dorsal root ganglion toxicity, among others.^63^ Notably, there has been a single case report of cardiac dysfunction, pericardial effusion, acute respiratory distress syndrome, and death within 8 days of infusion of a high-dose recombinant AAV packaged CRISPR gene engineering therapy in a patient with advanced muscular dystrophy.^128^ However, the mechanism of injury remains unclear as postmortem analysis showed no AAV antibodies or T-cell reactivity to suggest an immune-mediated response and minimal transgene expression in the liver. Yet, concern for CRISPR-mediated toxicity remains.

Beyond caring for patients who have undergone CRISPR treatments, anaesthetists might also be situated to implement CRISPR technology to treat critically ill patients. Sepsis and critical illness from infectious disease are leading causes of ICU admission worldwide.^129^^,^^130^ While initial resuscitative treatments of sepsis are standardised, identifying a responsible pathogen and starting appropriate treatment is often lifesaving. CRISPR can be repurposed for nucleic acid detection, and CRISPR diagnostic technology has been shown to rapidly and reliably identify pathogens from various sample types.^131^, ^132^, ^133^ Assays that rapidly identify pathogens in sepsis would be enormously beneficial, potentially allowing earlier diagnosis and treatment with antimicrobials.^134^^,^^135^ Finally, CRISPR technology potentially allows for real-time *in vivo* immune modulation during sepsis.

Recently, investigators used an AAV delivery system in mice to inhibit the Myd88 gene *in vivo* using CRISPR technology.^7^ Myd88 plays a critical role in modulating both innate and adaptive immune responses to pathogens. Specifically, Myd88 influences Toll-like receptor signalling pathways and high levels of Myd88 are associated with greater sepsis mortality.^136^ Investigators showed Myd88 inhibition provided preventive protection against sepsis in both Cas9 transgenic mice (mice with Cas9 engineered into their genome) and normal mice.^7^ Delivery of the CRISPRi system via LNP during sepsis was able to influence the course of sepsis.^7^ Inhibition of the Myd88 locus was associated with immunomodulation and downregulation of the tumour necrosis factor-alpha and ICAM-1 signalling pathways.^7^ Whether caring for a patient who received CRISPR therapy or using CRISPR technology to affect patient outcomes, understanding the role of CRISPR in the perioperative and critical care arenas is of increasing importance in anaesthesia.

### CRISPR in chronic pain

CRISPR therapy has great potential for treatment of chronic pain. Current pharmacotherapy for chronic pain can be limited by treatment adverse effects and patient tolerance. Novel pain targets have been identified by sequencing individuals and families with dysregulated pain or sensory signalling, most notably *SCN9A,* which encodes the sodium channel Nav1.7.^137^

Nav1.7 is one of three voltage-gated sodium channels expressed in primary afferent nociceptive neurones with key roles in pain signalling.^138^ Despite being an attractive target for chronic pain therapies, previous small molecule inhibitors of Nav1.7 have failed because of poor bioavailability and lack of specificity given the similarity of Nav subtypes.^139^ Antibody therapies against Nav1.7 have similarly failed as successful antibody binding has not translated into optimal sodium channel inhibition.^140^ Recently, CRISPR-dCas9 was used to epigenetically repress Nav1.7 *in vivo*.^6^ Administered via intrathecal lumbar injection using an AAV delivery system, this CRISPR-dCas9 epigenome engineering method effectively repressed Nav1.7 in dorsal root ganglia and durably reduced pain in three different models without impacting motor function or giving side-effects in mice.^6^ This preclinical work holds promise for the treatment of refractory chronic pain and demonstrates the potential of CRISPR epigenetic modulation as a therapeutic modality.

Another potential pain target for CRISPR is the fatty acid amide hydrolase (FAAH) chromosomal region.^137^^,^^141^ FAAH is the major catabolic enzyme for bioactive lipids involved in nociception, anxiety, and depression. Homozygous carriers of a common SNP in the *FAAH* gene have demonstrated reduced need for postoperative analgesics. Furthermore, recent work characterised a mutation in the human *FAAH* chromosomal region in a patient with lifelong insensitivity to pain and lack of anxiety.^141^^,^^142^ This long noncoding RNA (lncRNA) *FAAH-OUT* was found to have an epigenetic role in regulating *FAAH* expression via DNMT1-dependent DNA methylation. Recently, the CRISPR-Cas9 system has been used to edit this region in human cells *in vitro* leading to a 50% reduction in *FAAH* expression.^143^

While reducing expression of both Nav1.7 and FAAH has shown promise in pain therapy, further work is needed to demonstrate clinical efficacy. An important characteristic of CRISPR as it relates to chronic pain therapy is that anaesthetists are skilled in the delivery of a potential CRISPR therapy in a tissue-specific manner (Fig. 5b). Chronic pain procedures routinely use ultrasound and fluoroscopy to deliver mechanical, chemical, or heat therapy to nerves of interest, including use of devices that deliver heat or radiotherapy to a target site. Conceivably, these devices could be modified to enable direct CRISPR endonuclease and gRNA delivery to a nerve target with minimal risk of tissue toxicity. If such a therapy failed, standard of care nerve ablation could follow with little risk to the patient. Overall, there is considerable potential for CRISPR to revolutionise chronic pain management.

### CRISPR as a direct anticancer agent

CRISPR has shown tremendous promise for cancer therapy. The role of CRISPR in advancing immunotherapy both through CAR-T therapy and other cell therapies is of significant interest.^144^, ^145^, ^146^, ^147^ However, as CRISPR transitions from an *ex vivo* therapy to an *in vivo* therapy, its potential use as an anticancer agent becomes more tractable. Recently, we showed that a CRISPR system can act as an effective anticancer agent itself both *in vitro* and *in vivo*.^8^ This approach showed tumour killing in a patient-derived glioblastoma model that was highly effective irrespective of epigenetic status, tumour mutation burden, or chemotherapy resistance,^8^ and correlated with increased survival in a mouse model.^8^ This approach allows CRISPR gRNAs to be generated specific to the patient's tumour, specifically killing only patient glioblastoma cells.^8^ This was achieved by designing gRNAs against the unique genetic signatures in the cancer from prior chemoradiation therapies.^8^ This approach not only demonstrated that cancer-specific targeting was possible, but also reveals a mechanism to force such signatures to occur in other cancers by leveraging oncologic chemoradiation signatures resulting from standard of care cancer therapy. This work, performed in an anaesthesiology department, highlights a new domain for the specialty.

Delivery of CRISPR therapy to the central nervous system (CNS) is non-trivial, but as in chronic pain, anaesthesiologists are skilled in neuraxial administration. Intrathecal injections of CRISPR delivery vehicles could lead to minimally invasive clinically effective delivery to CNS tumours. This approach could also be used to target tumours outside the CNS. By using ultrasound technology, CRISPR therapy could be delivered in a highly localised manner to specific tumour sites (Fig. 5c). Finally, CRISPR using a dCas9 endonuclease fused to a bioluminescent marker with a gRNA that recognises tumour-specific mutations, could be used to visualise the margins of tumour enabling surgeons to get precise tumour margins on resection.^148^ In all these settings, anaesthesiologists can act as a conduit for CRISPR into medicine.

## CRISPR and anaesthesia

CRISPR therapy is now part of modern medicine and will affect patients cared for by anaesthetists in the operating theatre, ICU, and chronic pain clinic.^149^^,^^150^ A fundamental understanding of CRISPR, its therapeutic forms, and its potential complications will be essential for effective management of these patients. Anaesthesiology as a specialty is poised to be at the forefront of the CRISPR revolution. Anaesthetists are endowed with skills to manage life-threatening physiology and pathology. They can precisely deliver therapies to various areas in the body, and they interface with every field of medicine. As such anaesthetists are the ideal physicians to pioneer novel CRISPR medicines and delivery methods to the clinic. CRISPR technology could enable an entirely new field of anaesthesiology with the anaesthetists as the interventional CRISPR physicians. The opportunities for CRISPR in anaesthesia are great as it matures as a major branch of medicine.

## Authors’ contributions

Contributed to the conception and design of this work: all authors

Drafted the manuscript: ARP, OM, JSC

Helped draft the manuscript: JH, MAG

Critical review and final approval of the manuscript: all authors

## Declaration of Generative AI and AI-assisted technologies in the writing process

The authors did not use generative AI tools in the conception, writing, or citation of any works in this manuscript.

## Funding

10.13039/100005831Foundation for Anesthesia Education and Research Fellowship Grant (RFG-08-15-2023-Perez); US National 10.13039/501100000026Institute of Aging (5R38AG070171); 10.13039/100031461UCSF Department of Anesthesia and Perioperative Care.

## Declarations of interest

ARP is a cofounder, officer, and equity holder of Silico Therapeutics (San Jose, CA, USA). JSC is a cofounder, officer, and equity holder of Mammoth Biosciences (Brisbane, CA, USA). OM is the spouse of JSC.

## References

[1] Jansen R., Embden J.D., Gaastra W., Schouls L.M. (2002). Identification of genes that are associated with DNA repeats in prokaryotes. Mol Microbiol.

[2] Doudna J.A., Charpentier E. (2014). Genome editing. The new frontier of genome engineering with CRISPR-Cas9. Science.

[3] U.S. Food and Drug Administration (2023). https://www.fda.gov/news-events/press-announcements/fda-approves-first-gene-therapies-treat-patients-sickle-cell-disease.

[4] U.S. Food and Drug Administration (2024). https://www.fda.gov/vaccines-blood-biologics/casgevy.

[5] Schambach A., Buchholz C.J., Torres-Ruiz R. (2024). A new age of precision gene therapy. Lancet.

[6] Moreno A.M., Alemán F., Catroli G.F. (2021). Long-lasting analgesia via targeted in situ repression of Na _V_ 1.7 in mice. Sci Transl Med.

[7] Moghadam F., LeGraw R., Velazquez J.J. (2020). Synthetic immunomodulation with a CRISPR super-repressor in vivo. Nat Cell Biol.

[8] Tan I.L., Perez A.R., Lew R.J. (2023). Targeting the non-coding genome and temozolomide signature enables CRISPR-mediated glioma oncolysis. Cell Rep.

[9] (1996). Practice guidelines for perioperative transesophageal echocardiography. A report by the American society of anesthesiologists and the society of cardiovascular anesthesiologists task force on transesophageal echocardiography. Anesthesiology.

[10] Li Y.L., Wong D.T., Wei W., Liu J. (2006). A novel acoustic window for trans-oesophageal echocardiography by using a saline-filled endotracheal balloon. Br J Anaesth.

[11] Wen C.H., Berkman T., Li X. (2023). Effect of intrathecal NIS-lncRNA antisense oligonucleotides on neuropathic pain caused by nerve trauma, chemotherapy, or diabetes mellitus. Br J Anaesth.

[12] Casas-Arroyave F.D., Osorno-Upegui S.C., Zamudio-Burbano M.A. (2023). Therapeutic efficacy of intravenous lidocaine infusion compared with thoracic epidural analgesia in major abdominal surgery: a noninferiority randomised clinical trial. Br J Anaesth.

[13] Echeverry G., Fischer G.W., Mead E. (2019). Next generation of cancer treatments: chimeric antigen receptor t-cell therapy and its related toxicities: a review for perioperative physicians. Anesth Analg.

[14] Haseeb F., Tholouli E., Wilson A. (2022). Chimeric antigen receptor T-cell therapy in adults: management of toxicities and implications for critical care. BJA Educ.

[15] Castellanos J.G., Perez A.R., Perez R.K. (2020). Anaesthesiologists as translational scientists. Br J Anaesth.

[16] Barrangou R., Marraffini L.A. (2014). CRISPR-Cas systems: prokaryotes upgrade to adaptive immunity. Mol Cell.

[17] Nuñez J.K., Harrington L.B., Kranzusch P.J., Engelman A.N., Doudna J.A. (2015). Foreign DNA capture during CRISPR–Cas adaptive immunity. Nature.

[18] Makarova K.S., Wolf Y.I., Iranzo J. (2020). Evolutionary classification of CRISPR–Cas systems: a burst of class 2 and derived variants. Nat Rev Microbiol.

[19] Hsu P.D., Lander E.S., Zhang F. (2014). Development and applications of CRISPR-Cas9 for genome engineering. Cell.

[20] Pinilla-Redondo R., Russel J., Mayo-Muñoz D. (2022). CRISPR-Cas systems are widespread accessory elements across bacterial and archaeal plasmids. Nucleic Acids Res.

[21] Marraffini L.A., Sontheimer E.J. (2010). CRISPR interference: RNA-directed adaptive immunity in bacteria and archaea. Nat Rev Genet.

[22] Sternberg S.H., Richter H., Charpentier E., Qimron U. (2016). Adaptation in CRISPR-Cas systems. Mol Cell.

[23] Wiedenheft B., Lander G.C., Zhou K. (2011). Structures of the RNA-guided surveillance complex from a bacterial immune system. Nature.

[24] Brouns S.J., Jore M.M., Lundgren M. (2008). Small CRISPR RNAs guide antiviral defense in prokaryotes. Science.

[25] Deltcheva E., Chylinski K., Sharma C.M. (2011). CRISPR RNA maturation by trans-encoded small RNA and host factor RNase III. Nature.

[26] Nishimasu H., Ran F.A., Hsu P.D. (2014). Crystal structure of Cas9 in complex with guide RNA and Target DNA. Cell.

[27] Jinek M., Chylinski K., Fonfara I., Hauer M., Doudna J.A., Charpentier E. (2012). A programmable dual-RNA–guided DNA endonuclease in adaptive bacterial immunity. Science.

[28] Jinek M., East A., Cheng A., Lin S., Ma E., Doudna J. (2013). RNA-programmed genome editing in human cells. eLife.

[29] Sternberg S.H., Redding S., Jinek M., Greene E.C., Doudna J.A. (2014). DNA interrogation by the CRISPR RNA-guided endonuclease Cas9. Nature.

[30] Anders C., Niewoehner O., Duerst A., Jinek M. (2014). Structural basis of PAM-dependent target DNA recognition by the Cas9 endonuclease. Nature.

[31] Pacesa M., Lin C.H., Cléry A. (2022). Structural basis for Cas9 off-target activity. Cell.

[32] Chen J.S., Dagdas Y.S., Kleinstiver B.P. (2017). Enhanced proofreading governs CRISPR–Cas9 targeting accuracy. Nature.

[33] Xue C., Greene E.C. (2021). DNA repair pathway choices in CRISPR-Cas9-mediated genome editing. Trends Genet.

[34] Chang H.H.Y., Pannunzio N.R., Adachi N., Lieber M.R. (2017). Non-homologous DNA end joining and alternative pathways to double-strand break repair. Nat Rev Mol Cell Biol.

[35] Román-Rodríguez F.J., Ugalde L., Álvarez L. (2019). NHEJ-mediated repair of CRISPR-Cas9-induced DNA breaks efficiently corrects mutations in HSPCs from patients with Fanconi anemia. Cell Stem Cell.

[36] Truong L.N., Li Y., Shi L.Z. (2013). Microhomology-mediated End Joining and Homologous Recombination share the initial end resection step to repair DNA double-strand breaks in mammalian cells. Proc Natl Acad Sci.

[37] Chauhan V.P., Sharp P.A., Langer R. (2023). Altered DNA repair pathway engagement by engineered CRISPR-Cas9 nucleases. Proc Natl Acad Sci.

[38] Bock C., Datlinger P., Chardon F. (2022). High-content CRISPR screening. Nat Rev Methods Primers.

[39] Zuccaro M.V., Xu J., Mitchell C. (2020). Allele-specific chromosome removal after Cas9 cleavage in human embryos. Cell.

[40] Nambiar T.S., Billon P., Diedenhofen G. (2019). Stimulation of CRISPR-mediated homology-directed repair by an engineered RAD18 variant. Nat Commun.

[41] Her J., Bunting S.F. (2018). How cells ensure correct repair of DNA double-strand breaks. J Biol Chem.

[42] Heyer W.D., Ehmsen K.T., Liu J. (2010). Regulation of homologous recombination in eukaryotes. Annu Rev Genet.

[43] Mao Z., Bozzella M., Seluanov A., Gorbunova V. (2008). DNA repair by nonhomologous end joining and homologous recombination during cell cycle in human cells. Cell Cycle.

[44] Liu M., Rehman S., Tang X. (2019). Methodologies for improving HDR efficiency. Front Genet.

[45] Xu X., Qi L.S. (2019). A CRISPR–dCas toolbox for genetic engineering and synthetic biology. J Mol Biol.

[46] Cong L., Ran F.A., Cox D. (2013). Multiplex genome engineering using CRISPR/Cas systems. Science.

[47] Komor A.C., Kim Y.B., Packer M.S., Zuris J.A., Liu D.R. (2016). Programmable editing of a target base in genomic DNA without double-stranded DNA cleavage. Nature.

[48] Nishida K., Arazoe T., Yachie N. (2016). Targeted nucleotide editing using hybrid prokaryotic and vertebrate adaptive immune systems. Science.

[49] Gaudelli N.M., Komor A.C., Rees H.A. (2017). Programmable base editing of A•T to G•C in genomic DNA without DNA cleavage. Nature.

[50] Rees H.A., Liu D.R. (2018). Base editing: precision chemistry on the genome and transcriptome of living cells. Nat Rev Genet.

[51] Anzalone A.V., Koblan L.W., Liu D.R. (2020). Genome editing with CRISPR–Cas nucleases, base editors, transposases and prime. Nat Biotechnol.

[52] Anzalone A.V., Randolph P.B., Davis J.R. (2019). Search-and-replace genome editing without double-strand breaks or donor DNA. Nature.

[53] Villiger L., Joung J., Koblan L., Weissman J., Abudayyeh O.O., Gootenberg J.S. (2024). CRISPR technologies for genome, epigenome and transcriptome editing. Nat Rev Mol Cell Biol.

[54] Chen P.J., Liu D.R. (2023). Prime editing for precise and highly versatile genome manipulation. Nat Rev Genet.

[55] Gibney E.R., Nolan C.M. (2010). Epigenetics and gene expression. Heredity (Edinb).

[56] Perez-Pinera P., Kocak D.D., Vockley C.M. (2013). RNA-guided gene activation by CRISPR-Cas9–based transcription factors. Nat Methods.

[57] Gilbert L.A., Larson M.H., Morsut L. (2013). CRISPR-mediated modular RNA-guided regulation of transcription in eukaryotes. Cell.

[58] Nuñez J.K., Chen J., Pommier G.C. (2021). Genome-wide programmable transcriptional memory by CRISPR-based epigenome editing. Cell.

[59] Cox D.B.T., Gootenberg J.S., Abudayyeh O.O. (2017). RNA editing with CRISPR-Cas13. Science.

[60] Song J., Zhuang Y., Yi C. (2024). Programmable RNA base editing via targeted modifications. Nat Chem Biol.

[61] Muhuri M., Levy D.I., Schulz M., McCarty D., Gao G. (2022). Durability of transgene expression after rAAV gene therapy. Mol Ther.

[62] Ledford H. (2024). The immune system can sabotage gene therapies — can scientists rein it in?. Nature.

[63] Ertl H.C.J. (2022). Immunogenicity and toxicity of AAV gene therapy. Front Immunol.

[64] Miller T.M., Pestronk A., David W. (2013). An antisense oligonucleotide against SOD1 delivered intrathecally for patients with SOD1 familial amyotrophic lateral sclerosis: a phase 1, randomised, first-in-man study. Lancet Neurol.

[65] Bäckström E., Bonetti A., Johnsson P. (2024). Tissue pharmacokinetics of antisense oligonucleotides. Mol Ther Nucleic Acids.

[66] Jo S.J., Chae S.U., Lee C.B., Bae S.K. (2023). Clinical pharmacokinetics of approved RNA therapeutics. Int J Mol Sci.

[67] Li Y., Glass Z., Huang M., Chen Z.Y., Xu Q. (2020). Ex vivo cell-based CRISPR/Cas9 genome editing for therapeutic applications. Biomaterials.

[68] Kingwell K. (2023). First CRISPR therapy seeks landmark approval. Nat Rev Drug Discov.

[69] Macmillan C. (2023). https://www.yalemedicine.org/news/gene-therapies-sickle-cell-disease.

[70] CRISPR Therapeutics and Vertex Pharmaceuticals (2024). https://www.casgevyhcp.com/sites/default/files/hcp-treatment-journey.pdf.

[71] Gillmore J.D., Gane E., Taubel J. (2021). CRISPR-Cas9 in vivo gene editing for transthyretin amyloidosis. N Engl J Med.

[72] Macarrón Palacios A., Korus P., Wilkens B.G.C., Heshmatpour N., Patnaik S.R. (2024). Revolutionizing in vivo therapy with CRISPR/Cas genome editing: breakthroughs, opportunities and challenges. Front Genome.

[73] Behr M., Zhou J., Xu B., Zhang H. (2021). In vivo delivery of CRISPR-Cas9 therapeutics: progress and challenges. Acta Pharm Sin B.

[74] Longhurst H.J., Lindsay K., Petersen R.S. (2024). CRISPR-Cas9 in vivo gene editing of *KLKB1* for hereditary angioedema. N Engl J Med.

[75] Swetha K., Kotla N.G., Tunki L. (2023). Recent advances in the lipid nanoparticle-mediated delivery of mRNA vaccines. Vaccines (Basel).

[76] Kazemian P., Yu S.Y., Thomson S.B., Birkenshaw A., Leavitt B.R., Ross C.J.D. (2022). Lipid-nanoparticle-based delivery of CRISPR/Cas9 genome-editing components. Mol Pharm.

[77] (2023). Lipid nanoparticle-enabled gene editing in the lung via inhalation. Nat Biotechnol.

[78] Kenjo E., Hozumi H., Makita Y. (2021). Low immunogenicity of LNP allows repeated administrations of CRISPR-Cas9 mRNA into skeletal muscle in mice. Nat Commun.

[79] Asmamaw Mengstie M. (2022). Viral vectors for the in vivo delivery of CRISPR components: advances and challenges. Front Bioeng Biotechnol.

[80] Wang D, Zhang F, Gao G. CRISPR-based therapeutic genome editing: strategies and in vivo delivery by AAV vectors. Cell 202; 181: 136–150. 10.1016/j.cell.2020.03.023 PMC7236621 32243786

[81] Xu C.L., Ruan M.Z.C., Mahajan V.B., Tsang S.H. (2019). Viral delivery systems for CRISPR. Viruses.

[82] Chew W.L., Tabebordbar M., Cheng J.K. (2016). A multifunctional AAV–CRISPR–Cas9 and its host response. Nat Methods.

[83] Pacesa M., Pelea O., Jinek M. (2024). Past, present, and future of CRISPR genome editing technologies. Cell.

[84] Schmidt M.J., Gupta A., Bednarski C. (2021). Improved CRISPR genome editing using small highly active and specific engineered RNA-guided nucleases. Nat Commun.

[85] Pausch P., Al-Shayeb B., Bisom-Rapp E. (2020). CRISPR-CasΦ from huge phages is a hypercompact genome editor. Science.

[86] Kim D.Y., Lee J.M., Moon S.B. (2022). Efficient CRISPR editing with a hypercompact Cas12f1 and engineered guide RNAs delivered by adeno-associated virus. Nat Biotechnol.

[87] Wu T., Liu C., Zou S. (2023). An engineered hypercompact CRISPR-Cas12f system with boosted gene-editing activity. Nat Chem Biol.

[88] Harrington L.B., Burstein D., Chen J.S. (2018). Programmed DNA destruction by miniature CRISPR-Cas14 enzymes. Science.

[89] Guo C., Ma X., Gao F., Guo Y. (2023). Off-target effects in CRISPR/Cas9 gene editing. Front Bioeng Biotechnol.

[90] Höijer I., Emmanouilidou A., Östlund R. (2022). CRISPR-Cas9 induces large structural variants at on-target and off-target sites in vivo that segregate across generations. Nat Commun.

[91] Wang Q., Yang J., Zhong Z., Vanegas J.A., Gao X., Kolomeisky A.B. (2021). A general theoretical framework to design base editors with reduced bystander effects. Nat Commun.

[92] Chen L., Zhang S., Xue N. (2023). Engineering a precise adenine base editor with minimal bystander editing. Nat Chem Biol.

[93] Doman J.L., Raguram A., Newby G.A., Liu D.R. (2020). Evaluation and minimization of Cas9-independent off-target DNA editing by cytosine base editors. Nat Biotechnol.

[94] Tao J., Bauer D.E., Chiarle R. (2023). Assessing and advancing the safety of CRISPR-Cas tools: from DNA to RNA editing. Nat Commun.

[95] Sasaki-Honda M., Akatsuka K., Sawai T. (2023). Is epigenome editing non-inheritable? Implications for ethics and the regulation of human applications. Stem Cell Rep.

[96] Grünewald J., Zhou R., Garcia S.P. (2019). Transcriptome-wide off-target RNA editing induced by CRISPR-guided DNA base editors. Nature.

[97] Zhou C., Sun Y., Yan R. (2019). Off-target RNA mutation induced by DNA base editing and its elimination by mutagenesis. Nature.

[98] Yi Z., Qu L., Tang H. (2022). Engineered circular ADAR-recruiting RNAs increase the efficiency and fidelity of RNA editing in vitro and in vivo. Nat Biotechnol.

[99] Reautschnig P., Wahn N., Wettengel J. (2022). CLUSTER guide RNAs enable precise and efficient RNA editing with endogenous ADAR enzymes in vivo. Nat Biotechnol.

[100] Herring-Nicholas A., Dimig H., Roesing M.R., Josephs E.A. (2024). Selection of extended CRISPR RNAs with enhanced targeting and specificity. Commun Biol.

[101] Kang S.H., Lee W.J., An J.H. (2020). Prediction-based highly sensitive CRISPR off-target validation using target-specific DNA enrichment. Nat Commun.

[102] Perez A.R., Sala L., Perez R.K., Vidigal J.A. (2021). CSC software corrects off-target mediated gRNA depletion in CRISPR-Cas9 essentiality screens. Nat Commun.

[103] Pan X., Qu K., Yuan H. (2022). Massively targeted evaluation of therapeutic CRISPR off-targets in cells. Nat Commun.

[104] National Institutes of Health (US); Biological Sciences Curriculum Study (2007).

[105] Lessard S., Francioli L., Alfoldi J. (2017). Human genetic variation alters CRISPR-Cas9 on- and off-targeting specificity at therapeutically implicated loci. Proc Natl Acad Sci U S A.

[106] Frangoul H., Locatelli F., Sharma A. (2024). Exagamglogene autotemcel for severe sickle cell disease. N Engl J Med.

[107] European Medicines Agency (2023). https://www.ema.europa.eu/en/news/first-gene-editing-therapy-treat-beta-thalassemia-and-severe-sickle-cell-disease.

[108] Bauer D.E., Kamran S.C., Lessard S. (2013). An erythroid enhancer of BCL11A subject to genetic variation determines fetal hemoglobin level. Science.

[109] Vertex Pharmaceuticals (2024). https://www.casgevy.com/sickle-cell-disease/treatment-journey.

[110] Gina Kolata (2023). https://www.nytimes.com/2023/12/08/health/fda-sickle-cell-crispr.html.

[111] Satija B. (2023). Vertex/CRISPR price sickle cell disease gene therapy at $2.2, Mln. Reuters.

[112] Johnson K.M., Jiao B., Ramsey S.D., Bender M.A., Devine B., Basu A. (2023). Lifetime medical costs attributable to sickle cell disease among nonelderly individuals with commercial insurance. Blood Adv.

[113] Pritišanac E., Urlesberger B., Schwaberger B., Pichler G. (2021). Fetal hemoglobin and tissue oxygenation measured with near-infrared spectroscopy—a systematic qualitative review. Front Pediatr.

[114] Wang J.Y., Doudna J.A. (2023). CRISPR technology: a decade of genome editing is only the beginning. Science.

[115] Healey N. (2024). Next-generation CRISPR-based gene-editing therapies tested in clinical trials. Nat Med.

[116] Roberts R. (2024). CRISPR clinical trials to follow in 2024 and beyond. Synthego: The Bench.

[117] Innovative Genomics Institute (2024).

[118] MAGNITUDE (2024). A Phase 3 study of NTLA-2001 in participants with transthyretin amyloidosis with cardiomyopathy (ATTR-CM), trial ID NCT06128629. clinicaltrials.gov.

[119] CRISPR Therapeutics (2024).

[120] (2024). A study evaluating the safety and efficacy of BEAM-201 in relapsed/refractory T-cell acute lymphoblastic leukemia (T-ALL) or T-cell lymphoblastic lymphoma (T-LL), trial ID NCT05885464. clinicaltrials.gov.

[121] (2024). A safety and efficacy study evaluating CTX112 in subjects with relapsed or refractory B-cell malignancies, trial ID NCT05643742. clinicaltrials.gov.

[122] Naeem M., Alkhnbashi O.S. (2023). Current bioinformatics tools to optimize CRISPR/Cas9 experiments to reduce off-target effects. Int J Mol Sci.

[123] Bae S., Park J., Kim J.S. (2014). Cas-OFFinder: a fast and versatile algorithm that searches for potential off-target sites of Cas9 RNA-guided endonucleases. Bioinformatics.

[124] Chen Q., Chuai G., Zhang H. (2023). Genome-wide CRISPR off-target prediction and optimization using RNA-DNA interaction fingerprints. Nat Commun.

[125] Perez A.R., Pritykin Y., Vidigal J.A. (2017). GuideScan software for improved single and paired CRISPR guide RNA design. Nat Biotechnol.

[126] Yen A., Zappala Z., Fine R.S., Majarian T.D., Sripakdeevong P., Altshuler D. (2024). Specificity of CRISPR-Cas9 editing in exagamglogene autotemcel. N Engl J Med.

[127] Locatelli F., Lang P., Wall D. (2024). Exagamglogene autotemcel for transfusion-dependent β-thalassemia. N Engl J Med.

[128] Lek A., Wong B., Keeler A. (2023). Death after high-dose rAAV9 gene therapy in a patient with Duchenne’s muscular dystrophy. N Engl J Med.

[129] Society of Critical Care Medicine (2023). Critical care statistics. Soc Crit Care Med.

[130] De Backer D., Deutschman C.S., Hellman J. (2024). Surviving sepsis campaign research priorities 2023. Crit Care Med.

[131] Broughton J.P., Deng X., Yu G. (2020). CRISPR–Cas12-based detection of SARS-CoV-2. Nat Biotechnol.

[132] Gootenberg J.S., Abudayyeh O.O., Kellner M.J., Joung J., Collins J.J., Zhang F. (2018). Multiplexed and portable nucleic acid detection platform with Cas13, Cas12a, and Csm6. Science.

[133] Chen J.S., Ma E., Harrington L.B. (2018). CRISPR-Cas12a target binding unleashes indiscriminate single-stranded DNase activity. Science.

[134] Kasputis T., He Y., Ci Q., Chen J. (2024). On-site fluorescent detection of sepsis-inducing bacteria using a graphene-oxide CRISPR-Cas12a (GO-CRISPR) system. Anal Chem.

[135] Selvam K., Ahmad Najib M., Khalid M.F., Ozsoz M., Aziah I. (2022). CRISPR-Cas systems-based bacterial detection: a scoping review. Diagnostics (Basel).

[136] AbdAllah N.B., Toraih E.A., Al Ageeli E. (2021). MYD88, NFKB1, and IL6 transcripts overexpression are associated with poor outcomes and short survival in neonatal sepsis. Sci Rep.

[137] Cox J.J., Reimann F., Nicholas A.K. (2006). An SCN9A channelopathy causes congenital inability to experience pain. Nature.

[138] Castoro R., Simmons M., Ravi V. (2018). *SCN11A* Arg225Cys mutation causes nociceptive pain without detectable peripheral nerve pathology. Neurol Genet.

[139] Kingwell K. (April 10 2019). Nav1.7 withholds its pain potential. Nat Rev Drug Discov Advance Access published on..

[140] Hutchings C.J., Colussi P., Clark T.G. (2019). Ion channels as therapeutic antibody targets. MAbs.

[141] Habib A.M., Okorokov A.L., Hill M.N. (2019). Microdeletion in a FAAH pseudogene identified in a patient with high anandamide concentrations and pain insensitivity. Br J Anaesth.

[142] Cajanus K., Holmström E.J., Wessman M., Anttila V., Kaunisto M.A., Kalso E. (2016). Effect of endocannabinoid degradation on pain: role of FAAH polymorphisms in experimental and postoperative pain in women treated for breast cancer. Pain.

[143] Mikaeili H., Habib A.M., Yeung C.W. (2023). Molecular basis of *FAAH-OUT* -associated human pain insensitivity. Brain.

[144] Stadtmauer E.A., Fraietta J.A., Davis M.M. (2020). CRISPR-engineered T cells in patients with refractory cancer. Science.

[145] Katti A., Diaz B.J., Caragine C.M., Sanjana N.E., Dow L.E. (2022). CRISPR in cancer biology and therapy. Nat Rev Cancer.

[146] Wang G., Chow R.D., Bai Z. (2019). Multiplexed activation of endogenous genes by CRISPRa elicits potent antitumor immunity. Nat Immunol.

[147] Ren J., Liu X., Fang C., Jiang S., June C.H., Zhao Y. (2017). Multiplex genome editing to generate universal CAR T cells resistant to PD1 inhibition. Clin Cancer Res.

[148] Ma H., Naseri A., Reyes-Gutierrez P., Wolfe S.A., Zhang S., Pederson T. (2015). Multicolor CRISPR labeling of chromosomal loci in human cells. Proc Natl Acad Sci U S A.

[149] Simoneaux R., Shafer S.L. (2022). CRISPR/Cas9 genomic editing. ASA Monitor.

[150] Paranjpe R., Diggle C., Kaura V., Shaw M.A., Hopkins P. (2023). Investigating candidate modifier loci in malignant hyperthermia susceptibility using CRISPR/Cas gene editing. Br J Anaesth.

